# Diphtheria and the Serum Treatment

**Published:** 1899-02

**Authors:** 


### Diphtheria and the Serum Treatment.

The London *Lancet* in its issue of December 31, 1898, reviews, as is its wont, the matters of note that have occurred in the medical world during the year. Speaking on the subject of public health, and especially with reference to diphtheria, our contemporary makes the following remarks: "But little has as yet been achieved to diminish the prevalence and fatality of diphtheria, especially in large towns and cities, including the metropolis. Few who are conversant with the etiology of this disease now entertain any doubt that with the largely increased facilities for personal communication which are afforded to children at the age when they are most likely to be attacked by diphtheria, which are the outcome of attendance in elementary schools, there has been a marked increase in the amount of death from this disease. The majority of observers also feel confident that by reason of the aggregation of young children in schools diphtheria among them is apt at times to assume a character of special potency for spread. Some time since it was announced that the education department of the privy council intended to make inquiry into the subject, but no indication of any such investigation has as yet been apparent. . . . Whatever can be done reasonably by school restrictions ought to be carried out without further delay." There can be no doubt that the regulations in force in London and in the large towns of Great Britain generally, are by no means so well conceived or so effectively carried out as in the big centers of population in the United States. Diphtheria, chiefly among the young, has been rampant in London within the past year, and this has been in face of the universally conceded excellent sanitary condition of that city. One cause of the prevalence of the disease with children is undoubtedly that the medical supervision of the elementary schools is not so strict as it should be. Again, for reasons not far to seek, the serum treatment in the United Kingdom has not yielded the satisfactory results which have been gained from the use of diphtheria antitoxin in this country, and perhaps to a lesser degree in some of the European countries. A short time ago statistics were published comparing the rate of mortality after the use of antitoxin in London with that of Paris and Berlin, much to the disadvantage of the English city. In Germany from 1885 to 1894 there were 119,038 deaths from diphtheria or croup, the average number thus being 11,904 per annum. In 1895, when diphtheria antitoxin was first used on a considerable scale, the deaths went down to 7,266. The diphtheria death rate was 10.69 per 10,000 of the population in the preceding ten years, and only 5.4 in 1895, so that the mortality had fallen 49.48 per cent. The showing of the antitoxin treatment of diphtheria in the city of Chicago is still more remarkable, and, if statistics are worth anything at all, should convince the most skeptical of the worth of serum as a curative of the disease. The antitoxin treatment of diphtheria by the department of health of Chicago was begun, as in Germany, in 1895. During the twenty-six months that antitoxin has been used the department physicians have visited and examined 5,739 cases of reported diphtheria. Of this number the Klebs-Loeffler bacillus was found in 3,956 cases, and 3,822 were treated by the department, of which 3,763 recovered and 259 died, giving a death rate of 6.77 per cent. Prior to the introduction of antitoxin the mortality rate in Chicago was about 35 per cent. But the results recorded for the treatment in November last, according to the report of the Chicago department of health, are nothing short of marvelous. During November 163 reported cases of diphtheria were investigated; of these 98 were shown to be true diphtheria and antitoxin was used. In addition to these cases there were four remaining from the previous month, so that in all 102 cases were treated, with 97 recoveries, 3 deaths, and 2 remaining under treatment at the end of the month, affording the extremely low death rate of 3 per cent. Intubation was performed on ten occasions, and of the deaths two occurred among the intubations. Of late there have been signs of a certain antipathy in the minds of some medical men against the use of antitoxin for diphtheria, and doubt has been thrown upon its effectiveness. The statistics quoted above should, however, have the effect of removing any remaining scruples which may still exist against the serum treatment, as, even allowing that statistics cannot be wholly depended upon, it is an unquestionable fact that since the year 1895 (when it was generally adopted in America and Germany) there has been an extraordinary diminution in the mortality from diphtheria.—Editorial, *New York Medical Record.*

